# Analysis of the clinical characteristics of insulin autoimmune syndrome induced by exogenous insulin in diabetic patients

**DOI:** 10.1186/s13098-021-00658-z

**Published:** 2021-04-07

**Authors:** Zuojun Li, Dan Yi, Lijuan Zheng, Shiran Li, Weijin Fang, Chunjiang Wang

**Affiliations:** 1 Department of Pharmacy, The Third Xiangya Hospital, Central South University, No. 138 Tongzipo Road, YueLu District, Changsha, 410013 Hunan China; 2 Drug Clinical Trial Center, Zhuzhou Central Hospital, Zhuzhou, China; 3 Xiangya School of Pharmaceutical Science, Central South University, Changsha, China

**Keywords:** Insulin autoimmune syndrome, Exogenous insulin, Insulin autoantibodies, Hypoglycemia

## Abstract

**Background:**

The exact incidence, clinical features and uniform diagnostic criteria of exogenous insulin autoimmune syndrome (EIAS) are still unclear. The purpose of this study is to explore the clinical characteristics of EIAS and to provide a structural approach for clinical diagnosis, treatment and prevention.

**Methods:**

The literature on EIAS in Chinese and English from 1970 to 2020 was collected for retrospective analysis.

**Results:**

A total of 122 patients (33 males and 73 females) were included in the study with a median age of 67 years (range 14–86) and a median HbA1c of 7.7%. EIAS mainly occurred in type 2 diabetes mellitus patients using premixed insulin. Symptoms manifested were hypoglycemia in 86.54%, recurrent episodes of symptomatic hypoglycemia in 35.58%, nocturnal hypoglycemia along with daytime hyperglycemia in 21.15% and recurrent hypoglycemia after discontinued insulin in 64.43%. The onset of symptoms occurred at night, in the early morning or during fasting, ranging from a few days to 78 months after the administration of insulin. The mean blood glucose level during the hypoglycemic phase was 2.21 mmol/L (range 1–3.4), and the serum insulin levels were mainly ≥ 100 U/mL and were associated with low C-peptide levels (≤ 10 ng/ml). Insulin autoantibodies (IAAs) were positive in all EIAS patients. The 75-g extended oral glucose tolerance test (OGTT) mainly showed a diabetic curve. Pancreatic imaging was unremarkable. Withdrawal of insulin alone or combination of oral hypoglycemic agents or replacement of insulin formulations or with corticosteroid treatment eliminated hypoglycemia in a few days to 3 months. IAA turned negative in 6 months (median, range 1–12). No hypoglycemia episodes were observed at a median follow-up of 6 months (range 0.5–60).

**Conclusions:**

EIAS is an autoimmune disease caused by insulin-binding antibodies in susceptible subjects. Insulin antibodies change glucose dynamics and could increase the incidence of hypoglycemic episodes. Detection of insulin antibodies is the diagnostic test. Changing therapeutic modalities reduced the incidence of hypoglycemic episodes.

## Background

Insulin autoimmune syndrome (IAS) is a rare cause of hypoglycemia and is also known as Hirata’s disease, which was first described in 1970 [1]. Classic IAS is characterized by extremely high serum insulin concentrations, elevated insulin autoantibody (IAA) titers, no prior exposure to exogenous insulin, and no pathological abnormalities of the pancreatic islets and has numerous presentations, such as being triggered in some by food intake and fasting in others [2]. IAS can be spontaneous or triggered by exposure to viruses or drugs [3]. Among them, drugs containing sulfhydryl groups are among the important triggers, such as methimazole, lipoic acid, glutathione, and penicillamine [4].

In recent years, hypoglycemia induced by exogenous insulin in diabetic patients has also shown symptoms similar to IAS. Some scholars named it exogenous insulin autoimmune syndrome (EIAS) as a nonclassic IAS [5]. EIAS exists in the published literature in the form of case reports that are gradually increasing in number. However, the exact incidence, clinical features and uniform diagnostic criteria of EIAS are still unclear. The evidence of IAS induced by exogenous insulin treatment is limited to case reports. In this study, we collected patients with exogenous insulin-induced autoimmune hypoglycemia to summarize the clinical features, diagnostic tests, treatment and prevention.

## Methods

We searched the Wanfang Data, China National Knowledge Infrastructure (CNKI), and Chinese VIP databases, PubMed/Medline, Web of Knowledge, OVID, Elsevier, Springer Link, Embase and Cochrane Library in Chinese and English from 1970 to 2020 and collected case reports and case analyses of exogenous insulin-induced insulin antibody syndrome for inclusion as preliminary studies. We identified the literature regarding insulin antibody syndrome induced by exogenous insulin using the search terms “insulin antibody syndrome”, “exogenous insulin”, “autoimmune hypoglycemia”, “hypoglycemia” and “Hirata disease”.

Inclusion and exclusion criteria: We collected case reports and case analyses of exogenous insulin-induced IAS for inclusion as preliminary studies. All EIAS patients were diagnosed based on hypoglycemia and laboratory tests, such as serum insulin level, C-peptide concentration and IAA. Duplicate cases, mechanistic studies, animal studies, review articles, sulfhydryl drugs or other factors that induce IAS, articles with incomplete data and articles that were only abstracts or lacked a full text were excluded.

## Results

### Patients' criteria

We screened a total of 2372 studies. After careful review by the two independent authors, only 56 studies, including 122 patients (87 males and 35 females), were included in the study. The median age of the patients was 67 years (range 14–86), the median duration of diabetes was 10 years (range 0.25–42), and the median HbA1c was 7.7% (range 5.4–14.4) (Table 1). Among the patients reported in the literature, 119 patients (97.54%) were from Asia, 100 patients (90.91%) had type 2 diabetes mellitus (T2DM), and 6 patients (5.45%) had type 1 diabetes mellitus (T1DM). One patient (0.82%) simultaneously took clopidogrel, which may cause IAS. Eight patients (6.56%) had autoimmune diseases.

**Table 1:** General data of 122 patients reported in case series/reports

Parameter		Value
Age (122)^a^	Years	67 (14,86)^b^
Sex	Male	87/122 (71.31%)
	Female	35/122 (28.69%)
BMI (76)^a^	kg/m^2^	23 (18.4,33.8)^b^
HbA1c (101)^a^	%	7.7 (5.4,14.4)^b^
Region	China	105/122 (86.07%)
	Japan	12/122 (9.64%)
	India	1/122 (0.82%)
	Finland	1/122 (0.82%)
	Korea	1/122 (0.82%)
	USA	2/122 (1.64%)
Indication for insulin (110)^a^	T1DM	6/110 (5.45%)
	T2DM	100/110 (90.91%)
	LADA	3/110 (2.73%)
	Pancreatic exocrine disease*	1/110 (0.91%)
Course of diabetes (118)^a^	Years	10 (0.25,42)^b^
Concomitant sulfhydryl drugs	Clopidogrel	1/122 (0.82%)
Concomitant autoimmune diseases	Grave’s diseases, Hashimoto's thyroiditis, sjogren syndrome, dermatitis eczematosa, psoriasis, poliomyelitis, rheumatoid arthritis	8/122 (6.56%)
Insulin dosage (100)^a^	IU/day	26(8,95)^b^
Duration of insulin use (86)^a^	Months	24(0.13,288)^b^
Insulin administration time before hypoglycemia (48)^a^	Months	12(0.07,78)^b^
Insulin types (115)^a^	Bovine and porcine insulin (4)^a^	4/115 (3.48%)
	Neutral protamine Hagedorn	4/4 (100%)
	Insulin analogue (29)^a^	29/115 (25.22%)
	Aspart	18/29 (62.07%)
	Glargine	10/29 (34.48%)
	Lispro	5/29 (17.24%)
	Detemir	5/29 (17.24%)
	Glulisine	1/29 (3.45%)
	No data	1/29 (3.45%)
	Recombinant human insulin (19)^a^	19/115 (16.52%)
	Short-acting insulin	14/19 (73.68%)
	Intermediate-acting insulin	11/19 (57.89%)
	Premixed insulin (72)^a^	72/115 (62.61%)
	Premixed human insulin	36/72 (50.00%)
	Novolin 30R	15/36 (41.67%)
	Humulin70/30	14/36 (38.89%)
	Novolin 50R	4/36 (11.11%)
	Gansulin30R	2/36 (5.56%)
	Gansulin50R	1/36 (2.78%)
	Premixed insulin analogues	35/72 (48.61%)
	Aspart 30	32/35 (91.42%)
	Lispro 25	3/35 (8.57%)
	Premixed animal-derived insulin	1/72 (1.39%)
	Wan Sulin 30R**	1/1 (100%)

BMI, Body Mass Index; LADA, Latent Autoimmune Diabetes; T1DM, type 1 diabetes mellitus; T2DM, type 2 diabetes mellitus

^a^Represents the number of patients out of 122 on which information regarding this particular parameter was provided

^b^Median (minimum–maximum)

^*^Total pancreatectomy for pancreatic adenocarcinoma

^**^Wan Sulin 30R contained 30% porcine insulin and 70% protamine zinc porcine insulin, which was produced by Wanbang Biopharmaceuticals in China

### Administration of insulin

The administration of insulin is shown in Table 1. The median daily dose of insulin was 26 IU (range 8–95). The median time of insulin was 12 months (range 0.07–78) before the onset of hypoglycemia. EIAS occurred in 72 patients (62.61%) using premixed insulin, 29 patients (25.22%) using insulin analogs, 19 patients (16.52%) using recombinant human insulin, and 4 patients (3.48%) using animal-derived insulin. Among 72 patients using premixed insulin, 36 patients (50.00%) and 35 patients (48.61%) used premixed human insulin and premixed insulin analogs, respectively. In patients with EIAS caused by insulin analogs, insulin aspart in 18 patients (62.07%) and glargine in 10 patients (34.48%) were the most common insulin analogs, followed by insulin detemir and insulin lispro in 5 patients (17.24%). Fourteen patients (73.68%) given short-acting human insulin and 11 patients (57.89%) given intermediate-acting human insulin were associated with EIAS induced by recombinant human insulin. Premixed insulin-related IAS most often occurred in 15 patients (41.67%) using Novolin 30R, 14 patients (38.89%) using Humulin 70/30 and 32 patients (91.42%) using Aspart 30.

### Clinical presentation

The clinical manifestations of EIAS in 104 patients (85.25%) are described in Table 2. EIAS mainly manifested as hypoglycemia in 90 patients (86.54%) and also manifested as hyperglycemia in 27 patients (30.00%) or without any symptoms of hypoglycemia in 4 patients (3.85%). Eleven patients (10.58%) with EIAS were also hospitalized with hyperglycemia, and even 4 patients were hospitalized with ketoacidosis (36.36%). Fourteen patients (13.46%) with EIAS also had allergic reactions that were simultaneous with the occurrence of hypoglycemia, which manifested as rash, pruritus, redness and swelling. Hypoglycemia mainly manifested as symptoms of the autonomic nervous system in 48 patients (53.33%), including palpitations, sweating, hunger and hand tremors, followed by neurological hypoglycemia in 27 patients (30.00%), including dizziness, coma, blurred consciousness, anxiety, fear, abnormal behavior and incontinence. In addition, recurrent episodes of symptomatic hypoglycemia occurred in 37 patients (35.58%), and nocturnal hypoglycemia along with daytime hyperglycemia occurred in 22 patients (21.15%). Sixty-seven patients (64.43%) still had recurrent hypoglycemia after insulin was discontinued. The time of onset of hypoglycemia was largely at night and in the early morning and during fasting in 68 patients (65.06%), before meals in 16 patients (18.60%) and 3 to 5 h after meals in 9 patients (10.47%).

**Table 2:** Clinical information on the122 included patients

Parameter		Value
Clinical manifestations (104)^a^	Hypoglycemia	90/104 (86.54%)
	Autonomic nervous system: palpitations, sweating, hunger and hand tremors	48/90 (53.33%)
	Neurological hypoglycemia: dizziness, coma, blurred consciousness, anxiety, fear, abnormal behavior and incontinence	27/90 (30.00%)
	Hypoglycemia	11/104 (10.58%)
	Ketoacidosis	4/11 (36.36%)
	No symptoms of hypoglycemia	4/104 (3.85%)
	Allergic reaction: rash, pruritus, redness and swelling	14/104 (13.46%)
	Recurrent episodes of symptomatic hypoglycemia	37/104 (35.58%)
	Nocturnal hypoglycemia along with daytime hyperglycemia	22/104 (21.15%)
	Recurrent hypoglycemia after discontinued insulin	67/104 (64.43%)
Onset time of hypoglycemia (86)^a^	Before meal	16/86 (18.60%)
	Night and early morning and fasting	68/86 (70.07%)
	3 to 5 h after meal	9/86 (10.47%)
Blood glucose (mmol/L)	Mean blood glucose during hypoglycemia (70)^a^	2.21 (1,3.4)b
	≤ 1	1/70 (1.43%)
	1–2	32/70 (45.71%)
	2–3	34/70 (47.57%)
	3– 4	3/70 (4.29%)
	Fasting blood glucose (80)^a^	5.59(1.24,13.9)^b^
Insulin (5–25 mU/L)	Insulin levels during hypoglycemia (44)^a^	
	50–100	2/44 (4.55%)
	100–300	12/44 (27.27%)
	300–1000	12/44 (27.27%)
	≥ 1000	18/44 (40.91%)
	Fasting insulin levels (86)^a^	
	50–100	11/86 (12.79%)
	100–300	20/86 (23.26%)
	300–1000	40/86 (46.51%)
	≥ 1000	15/86 (17.44%)
C-peptide (1.1–4.4 ng/ml)	C-peptide levels during hypoglycemia (39)^a^	
	< 5	21/39 (53.85%)
	5–10	13/39 (33.33%)
	10–50	5/39 (12.82%)
	Fasting C-peptide levels (87)^a^	
	< 5	57/87 (65.52%)
	5–10	18/87 (20.69%)
	≥ 10	12/87 (13.79%)
Antibody		
IAA (122)^a^	Positive	122/122 (100%)
ICA (85)^a^	Positive	8/85 (9.41%)
GADA (89)^a^	Positive	7/89 (7.87%)
OGTT (65)^a^	IGT	6/65 (9.23%)
	Diabetic curve	59/65 (90.77%)
	3-to 5-h hypoglycemia	14/39 (35.60%)

CT, computed tomography; GADA, Glutamic Acid Decarboxylase Antibody; ICA, Islet Cell Autoantibody; IGT, impaired glucose tolerance; OGTT, oral glucose tolerance test

^a^Represents the number of patients out of 106 on which information regarding this particular parameter was provided

^b^Mean (minimum–maximum)

### Laboratory tests

The laboratory test results related to EIAS are summarized in Table 2. The mean blood glucose level at the onset of hypoglycemia was 2.21 mmol/L (range 1–3.4), and 66 patients (94.29%) had blood glucose levels of ≤ 3 mmol/L during hypoglycemic episodes. The serum insulin levels were ≥ 1000 U/mL in 18 patients (40.91%) during the onset of hypoglycemia and 100–1000 U/ml in 42 patients (95.45%). C-peptide was < 10 ng/ml in 34 patients (87.18%). The mean fasting blood glucose level was 5.59 mmol/L (1.24, 13.9) in 80 patients, accompanied by fasting insulin levels ≥ 100 U/mL in 75 patients (87.21%), and fasting C-peptide levels were ≤ 10 ng/ml in 75 patients (86.21%). All 122 EIAS patients were positive for IAA, 8 patients (9.41%) were ICA positive, and 9 patients (7.87%) were glutamic acid decarboxylase antibody (GADA) positive. Seven patients (5.74%) employed a continuous glucose monitor (CGM).

The 75-g extended oral glucose tolerance test (OGTT) showed diabetic curves in 59 patients (90.77%), impaired glucose tolerance (IGT) in 6 patients (9.23%), and 3- to 5-h hypoglycemia in 14 patients (35.60%). Nine patients underwent histocompatibility leukocyte antigen (HLA) genotype testing, of whom 7 patients were related to HLA-DR4 and 4 patients were related to HLA-DQ3. HLA-DR4 showed DRB1*0405/1302, DRB1*0405/080302, DRB1*0406, DRB1*080302/090102 and HLA-DRB1*0406/040101. HLA-DQ3 showed DQB1*0401/*0604, DQA1*0302-DQB1*0302/0303, DQA1*0601/0102-DQB1*0604 and DQB1*030201/050101.

### Imaging examination

The imaging examination of EIAS patients mainly included pancreas computed tomography (CT) in 50 patients, magnetic resonance imaging (MRI) in 9 patients and abdominal ultrasound in 14 patients (Table 3). CT scans showed normal pancreas in 48 EIAS patients (96%) and focal cystic change and slightly full pancreatic head in 1 patient (2%). Both MRI and ultrasound showed a normal pancreas in EIAS patients.

**Table 3:** Imaging examination, treatment and prognosis of 122 EIAS patients reported in case series/reports

Parameter		Value
Imaging examination		
CT (50)^a^	Normal	48/50 (96%)
	Focal cystic change	1/50 (2%)
	Slightly full pancreatic head	1/50 (2%)
MRI (9)^a^	Normal	9/9 (100%)
Ultrasound (14)^a^	Normal	14/14 (100%)
Therapy (122)^a^	Discontinued insulin	122/122 (100%)
	Switch to oral hypoglycemic drugs	68/122 (55.74%)
	Switch to another insulin type	33/122 (27.05%)
	Lifestyle modification	20/122 (16.39%)
	Medical Nutrition therapy	1/122 (0.82%)
	Corticosteroids	39/122 (31.97%)
	Plasmapheresis	4/122 (3.28%)
Hypoglycemia disappearance time (45)^a^	Corticosteroids (Months)	1 (0.07,6)^b^
	Non-corticosteroids (Months)	2 (0.03,8)^b^
IAA turn negative time (20)^a^	Months	6 (1, 12)^b^
Follow-up time (55)^a^	Months	6 (0.5,60)^b^

CT, computed tomography; MRI, magnetic resonance imaging;

^a^Median (minimum–maximum)

^b^Represents the number of patients out of 122 on which information regarding this particular parameter was provided

### Treatment and prognosis

A total of 122 patients (100%) eventually stopped receiving EIAS-related exogenous insulin and were given small, frequent meals and low-carbohydrate treatment (Table 3). A total of 68 patients (55.74%) switched to oral hypoglycemic drugs, 33 patients (27.05%) switched to other insulin types, 20 patients (16.39%) adopted lifestyles without hypoglycemic drugs, 39 patients (31.97%) were given glucocorticoid therapy, and 4 patients (3.28%) were treated with plasmapheresis. Interestingly, 1 patient was given medical nutrition therapy for EISA and achieved good clinical effects [6]. The median times to disappearance of hypoglycemia were 1 month (range 0.07–6) in the corticosteroid treatment group and 2 months (range 0.03–8) in the noncorticosteroid treatment group. The median time for IAA to become negative was 6 months (range 1–12). None of the patients had EIAS-related hypoglycemia episodes at a median follow-up of 6 months (range 0.5–60).

## Discussion

Hypoglycemia is the most common acute complication of diabetic patients receiving antidiabetic therapies, especially those receiving insulin therapies, and can be easily improved by adjusting the insulin dose or improving one’s lifestyle. In recent years, the incidence of autoimmune hypoglycemia has gradually increased due to increased administration of exogenous insulin. The primary diagnostic criteria are the presence of insulin-binding antibodies, high insulin and low C-peptide [7], although these criteria are not universally accepted [2].

Our research showed that EIAS may occur in diabetic patients using various types of insulin, especially T2DM patients who use premixed insulin human insulin and premix analogs. HbA1c, which represents 2-month glycemia, was usually > 7% in EIAS patients [8]. The condition occurred 2 days to 78 months after insulin administration. Patients with EIAS mainly showed symptoms of the autonomic nervous system, such as palpitations, sweating, and hunger. Some patients may have skin allergic reactions at the same time during episodes of hypoglycemia. Few patients present with severe central system dysfuntion may present with central nervous system dysfunction, such as dizziness, coma, blurred consciousness, anxiety, fear, abnormal behavior and incontinence. Blood glucose fluctuation was the main clinical manifestation of EIAS, which can manifest as recurrent hypoglycemia, and nocturnal hypoglycemia along with daytime hyperglycemia. There were still recurrent episodes of hypoglycemia after stopping exogenous insulin for longer than 5 half-lives. Blood glucose levels generally ranged from 1 to 3 mmol/L at the time of hypoglycemia. The OGTT is usually used to determine the dynamic changes of glucose, insulin and C-peptide fluctuations in the reaction [9]. The OGTT showed normal fasting blood glucose (~ 5.59 mmol/L), insulin and C peptide aberration. EIAS patients usually have a higher serum insulin concentration, and their C-peptide and proinsulin levels may or may not increase. Many scholars recommend measuring the ratio of insulin to C-peptide to diagnose IAS [2].

IAS is caused by a large amount of IAA against insulin and/or proinsulin in the circulation [3, 10]. IAA is the first-line test to determine the cause of hypoglycemia in nondiabetic adults in the recently published clinical practice guidelines by the American Endocrine Society [11]. The predominant immunoglobulin of IAA is IgG; other immunoglobulins, such as IgM, IgA and IgE, have also been reported [12]. IAA can be divided into low affinity/high capacity and high affinity/low capacity [13]. The presence of such endogenous binding antibodies distorts glucose metabolism in states of hyper- and hypoglycemia. The severity and duration of hypoglycemia and the swing from a hyperglycemic to a hypoglycemic state are closely related to the binding characteristics of insulin to IAA i.e. intrinsic dissociation rate constant (K-1), titer and intrinsic affinity/capacity [3]. Although the presence of insulin-binding antibodies is a common feature of all IAS, some predominantly bind proinsulin and contribute to raising insulin levels by cross-reacting. However, proinsulin has only a tenth of the bioactivity of insulin, and symptoms in some cases are usually milder [3]. Elevated blood glucose levels and diet can stimulate pancreatic β-cells to produce insulin, which is bound by IAA, thereby rendering insulin ineffective, forming a "reservoir" and causing postprandial hyperglycemia. Then, insulin/proinsulin dissociates from these endogenous antibodies, giving rise to hypoglycemia [14]. However, EIAS is caused by IAA against exogenous insulin, which can bind endogenous and exogenous insulin and cause poor glycemic control and hypoglycemia by a mechanism similar to IAS. The condition may be related to insulin purity, insulin molecular structure, polymerization status, delivery route of insulin, patient immune status, and genetic susceptibility, among other factors [15–19].

Genetic susceptibility plays an important role in the pathogenesis of IAS, especially HLA-B15, HLA-DR4 and HLA-DR7. Eastern Asian patients with a high prevalence of DRB1*0406 are therefore at higher risk of developing IAS than populations with DRB1*0403 and DRB1*0407, whereas Caucasian IAS patients mainly express HLA-DRB1*0403 [20, 21].

Although several therapeutic approaches to EIAS have been reported, the optimal treatment is yet to be recommended. Patients with mild EIAS can significantly improve their hypoglycemic symptoms by lifestyle intervention and stopping related insulin. Changing therapeutic modalities can help reduce hypoglycemic episodes (note that bovine/porcine insulin and insulin analogs could trigger EIAS) [22]. Treatment of patients with EIAS includes glucocorticoids, immunoglobulin infusions or immunosuppressive agents such as mycophenolate mofetil, cyclophosphamide and rituximab, and plasmapheresis therapy can be performed if necessary [23–26]. Recently, medical nutrition therapy was effective in the management of hypoglycemia induced by EIAS and provided a new idea for the treatment of EIAS [6]. A CGM may be an effective treatment for IAS or EIAS. CGMs have been successfully used in IAS refractory to steroids, and flash glucose monitoring (FGM) has been successfully used in α-lipoic acid-induced IAS [27, 28].

EIAS is a self-limiting disease and hypoglycemia can subside and disappear within a few months after discontinuing the related insulin. Patients with EIAS did not have recurrent hypoglycemia during the follow-up period with or without corticosteroids. IAA became negative from 1 to 12 months after the discontinuation of insulin due to the half-life of immunoglobulin antibodies (3–4 weeks). Existing studies have found that IAS and EIAS have no significant differences in the remission rate of hypoglycemia, treatment strategies and prognosis, while persistent hypoglycemia occurs in IAS patients and alternating hypoglycemia and hyperglycemia occurs in most EIAS patients, similar to our research [29]. Defining the difference between IAS and EIAS still requires more research.

## Conclusions

EIAS should be considered in poorly controlled diabetic patients receiving any form of insulin. The single most important diagnostic test is a high concentration of insulin antibodies. Antibody characteristics, which vary from patient to patient, determine the impact on glucose metabolism and dynamics. Changing therapeutic modalities could help reduce hypoglycemic episodes.

## Data Availability

The datasets used and/or analysed during the current study are available from the corresponding author on reasonable request.

## Abbreviations

IAS: Insulin autoimmune syndrome
EIAS: Exogenous insulin autoimmune syndrome
MRI: Magnetic resonance imaging
OGTT: Oral glucose tolerance
GADA: Glutamic Acid Decarboxylase Antibody
CT: Computed tomography
HL: Histocompatibility leukocyte antigen
IGT: Impaired glucose tolerance
IAA: Insulin autoantibody
CGM: Continuous glucose monitor
FGM: Flash glucose monitoring
